# Supplementary material for: DLK-MAPK Signaling Coupled with DNA Damage Promotes Intrinsic Neurotoxicity Associated with Non-Mutated Tau
Source: Mol Neurobiol. 2023 Nov 13;61(5):2978–95. doi: 10.1007/s12035-023-03720-1 (PMC11043018; doi:10.1007/s12035-023-03720-1)
Supplement: Supplementary file 1 — (DOCX 2394 kb) [file 12035_2023_3720_MOESM1_ESM.docx]

**Supplemental figures and legends**

**
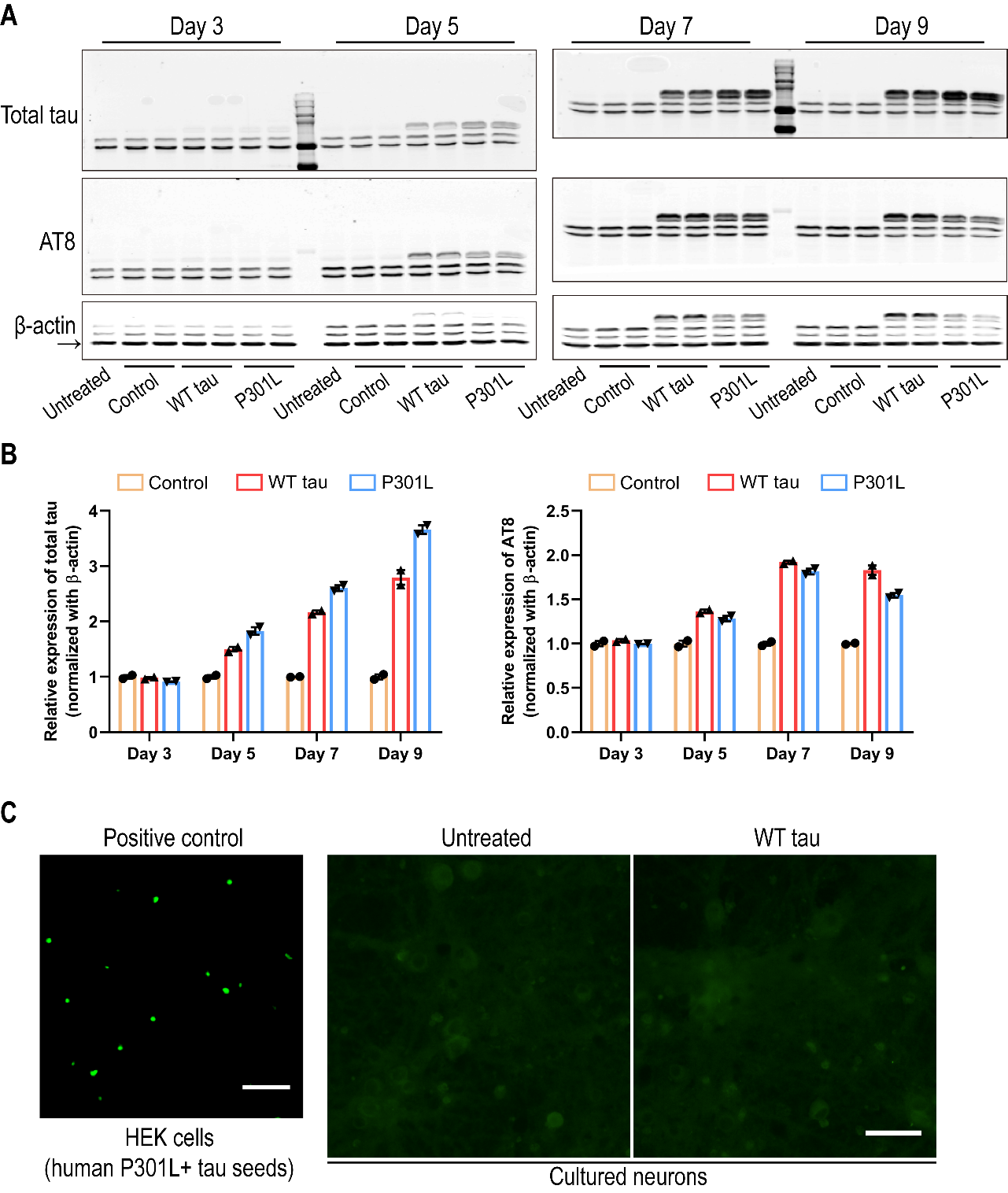
**

**Figure S1. Expression of human tau proteins in mouse primary neurons.**

**(A)** Expression of total and phosphorylated tau was evaluated by western blot in untreated, control and WT tau groups at different time points after AAV infection.

(**B)** Quantification of total and phosphorylated tau from **A**. All groups were normalized to the endogenous mouse tau in control group.

**(C)** Thioflavin-S staining of untreated and WT tau groups. HEK cells, which transfected with a human P301L tau plasmid and treated with tau seeds, were used as a positive control. The scale bar represents 50 μm.

**
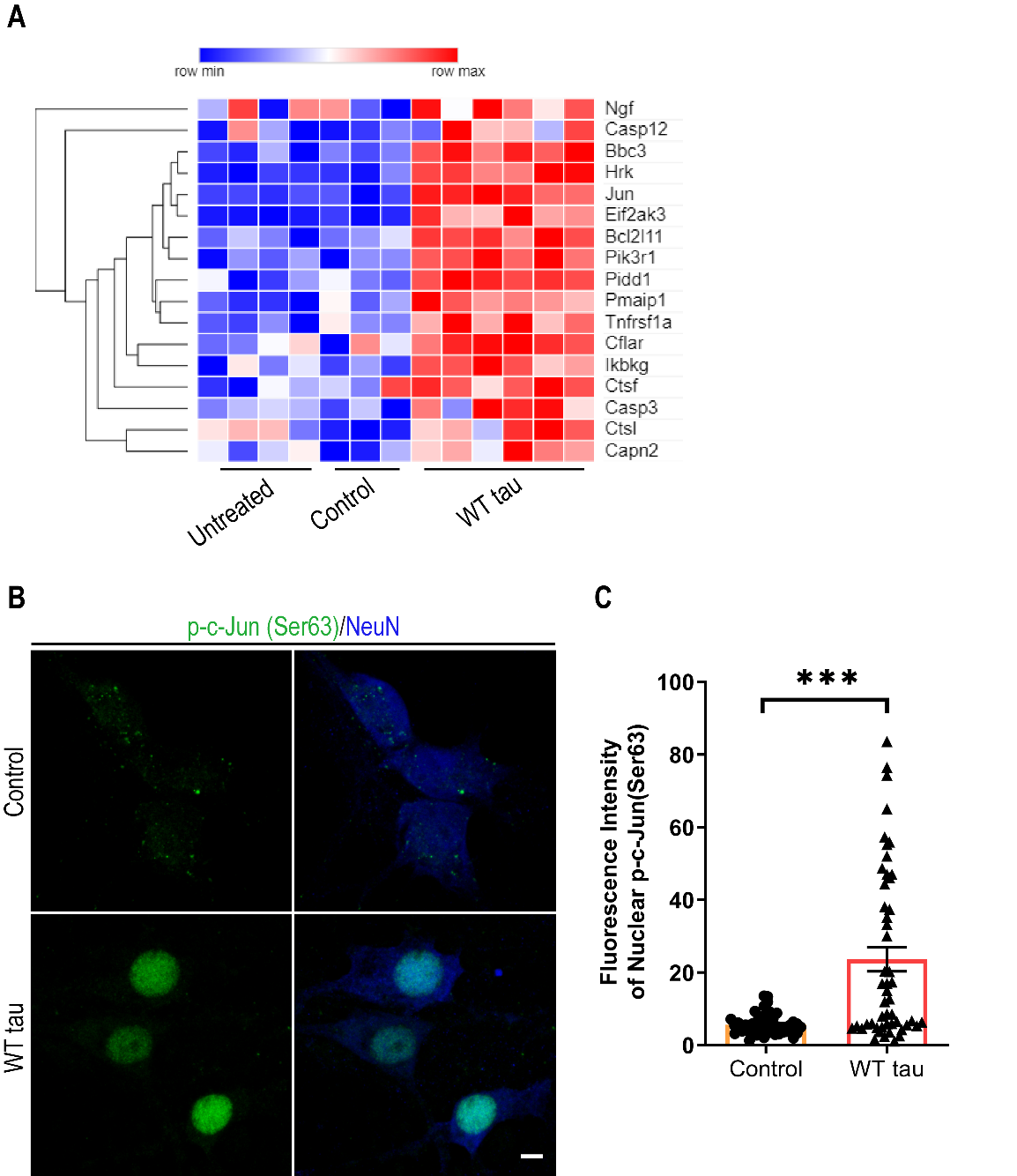
**

**Figure S2. Expression of apoptotic genes and activation of c-Jun in WT hTau-expressing neurons.**

**(A)** Heatmap showing DEGs recruited in cell apoptosis pathways in untreated, control, WT tau groups. The rows in heatmap were sorted by similarity via hierarchical clustering.

**(B)** Representative images of p-c-Jun (Ser63) staining in control and WT tau groups. The scale bar represents 5 μm.

**(C)** Quantification of nuclear p-c-Jun (Ser63) signals in cultures as shown in **B**. n=49, 50 neurons in control and WT tau groups, respectively.

Data are presented as mean ± SEM. Statistical significance was determined using unpaired, two-tailed Student’s t test in C.

**
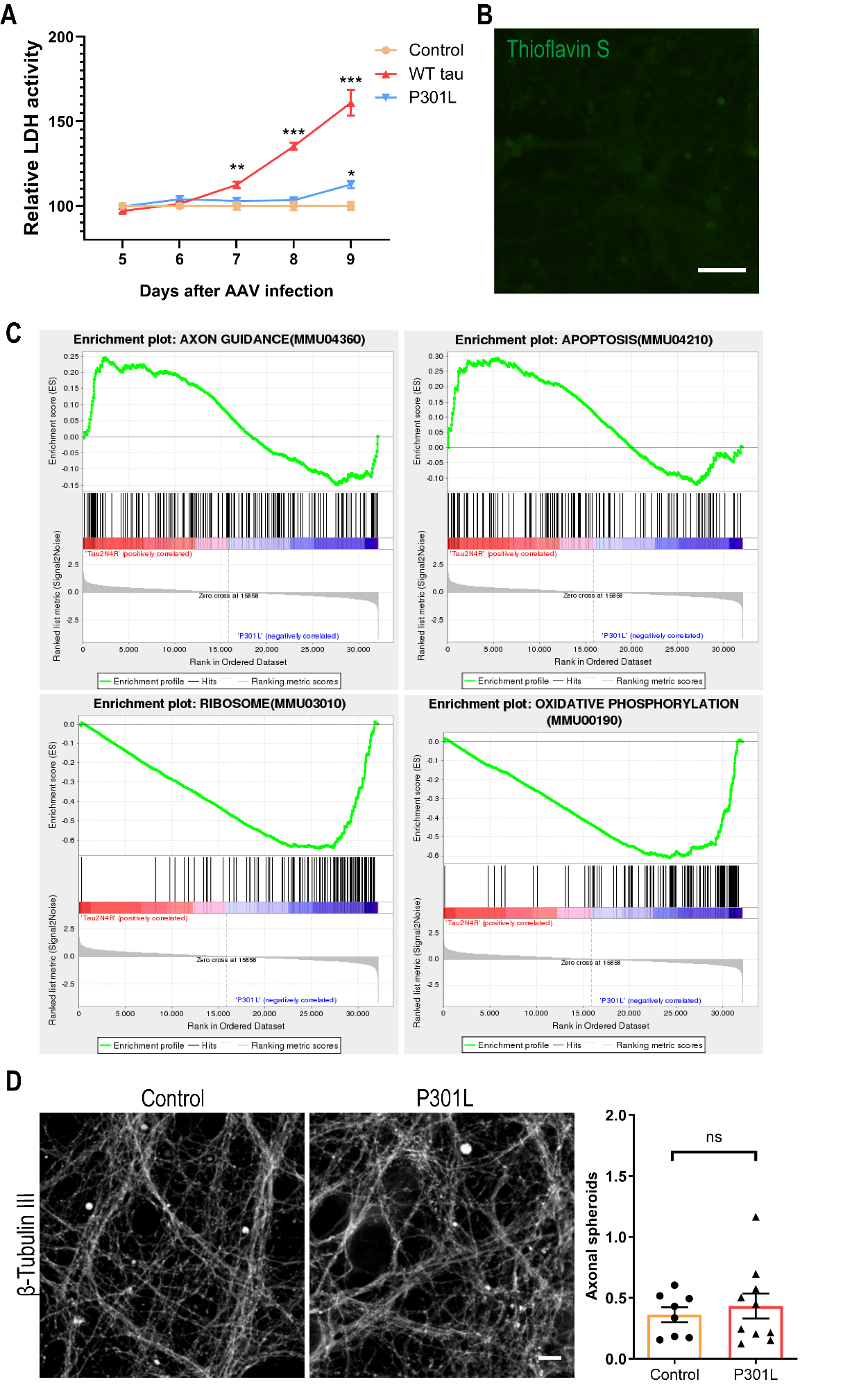
**

**Figure S3. Differential effects by P301L and WT hTau on primary neurons.**

**(A)** Kinetics of LDH activity in culture media of control, WT tau and P301L tau groups. n=6-8 wells/group.

**(B)** Thioflavin-S staining of P301L hTau-expressing neurons. The scale bar represents 50 μm.

**(C)** The enrichment plots showing differential association of axon guidance, apoptosis, ribosome and oxidative phosphorylation pathways with WT and P301L tau.

**(D)** Representative images of β-tubulin III staining in control and P301L groups at day 7 post-infection and quantification of axonal spheroids. The scale bar represents 5 μm. n=8, 10 individual images in control, P301L groups, respectively.

Data are presented as mean ± SEM. Statistical significance was determined using one-way ANOVA with Tukey’s multiple comparisons test in A, or unpaired, two-tailed Student’s t test in D.

**
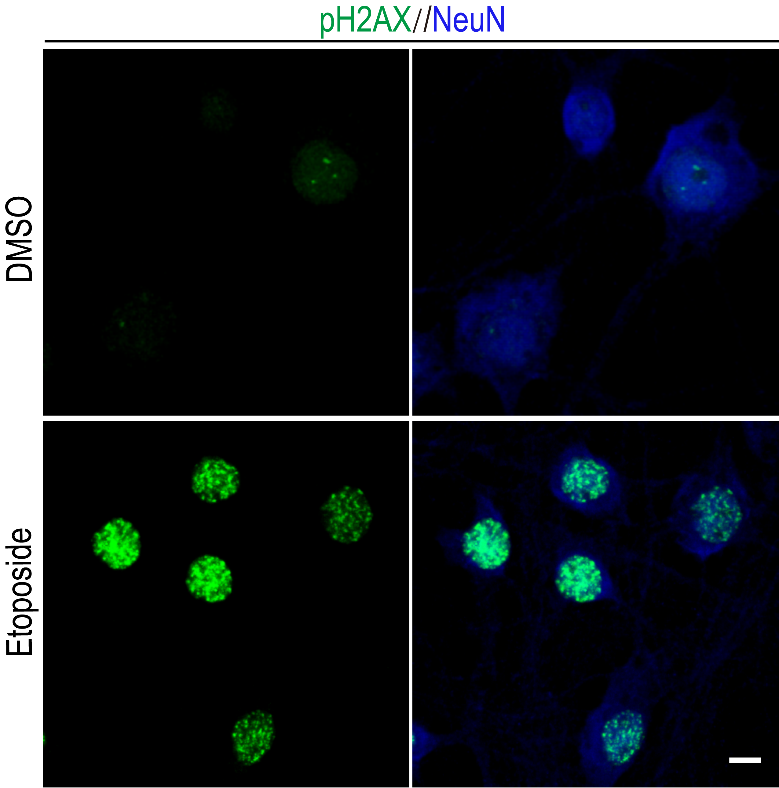
**

**Figure S4. Etoposide-induced DNA damage response.**

Representative images of pH2AX staining in DMSO- or etoposide-treated groups. The scale bar represents 5 μm.

**Supplementary Information**

Supplemental data S1. Expression data reported in Fig 5A

Supplemental data S2. Expression data reported in Fig 5B and 6H

Supplemental data S3. Expression data reported in Fig 6G

Supplemental data S4. Sequence of AAV constructs

**Table S1. Primer sequence pairs used for quantitative real-time PCR.**

| **Gene** | **Forward Sequence** | **Reverse Sequence** |
| --- | --- | --- |
| ***Hprt1*** | CTGGTGAAAAGGACCTCTCGAAG | CCAGTTTCACTAATGACACAAACG |
| ***Casp3*** | GGAGTCTGACTGGAAAGCCGAA | CTTCTGGCAAGCCATCTCCTCA |
| ***Bbc3*** | ACCGCTCCACCTGCCGTCAC | ACGGGCGACTCTAAGTGCTGC |
| ***Cdkn1a*** | TCGCTGTCTTGCACTCTGGTGT | CCAATCTGCGCTTGGAGTGATAG |
| ***Cdkn1b*** | AGCAGTGTCCAGGGATGAGGAA | TTCTTGGGCGTCTGCTCCACAG |
