# Supplementary material for: DLK-MAPK Signaling Coupled with DNA Damage Promotes Intrinsic Neurotoxicity Associated with Non-Mutated Tau
Source: Mol Neurobiol. 2023 Nov 13;61(5):2978–95. doi: 10.1007/s12035-023-03720-1 (PMC11043018; doi:10.1007/s12035-023-03720-1)
Supplement: Supplementary file 5 — (PDF 18 kb) [file 12035_2023_3720_MOESM5_ESM.pdf]

Wild-type Tau 2N4R

ATGGCTGAGCCCCGCCAGGAGTTCGAAGTGATGGAAGATCACGCTGGGACGTACGG
GTTGGGGGACAGGAAAGATCAGGGGGGCTACACCATGCACCAAGACCAAGAGGGT
GACACGGACGCTGGCCTGAAAGAATCTCCCCTGCAGACCCCCACTGAGGACGGATC
TGAGGAACCGGGCTCTGAAACCTCTGATGCTAAGAGCACTCCAACAGCGGAAGATG
TGACAGCACCCTTAGTGGATGAGGGAGCTCCCGGCAAGCAGGCTGCCGCGCAGCCC
CACACGGAGATCCCAGAAGGAACCACAGCTGAAGAAGCAGGCATTGGAGACACCC
CCAGCCTGGAAGACGAAGCTGCTGGTCACGTGACCCAAGCTCGCATGGTCAGTAAA
AGCAAAGACGGGACTGGAAGCGATGACAAAAAAGCCAAGGGGGCTGATGGTAAAA
CGAAGATCGCCACACCGCGGGGAGCAGCCCCTCCAGGCCAGAAGGGCCAGGCCAA
CGCCACCAGGATTCCAGCAAAAACCCCGCCCGCTCCAAAGACACCACCCAGCTCTG
GTGAACCTCCAAAATCAGGGGATCGCAGCGGCTACAGCAGCCCCGGCTCCCCAGGC
ACTCCCGGCAGCCGCTCCCGCACCCCGTCCCTTCCAACCCCACCCACCCGGGAGCCC
AAGAAGGTGGCAGTGGTCCGTACTCCACCCAAGTCGCCGTCTTCCGCCAAGAGCCG
CCTGCAGACAGCCCCCGTGCCCATGCCAGACCTGAAGAATGTCAAGTCCAAGATCG
GCTCCACTGAGAACCTGAAGCACCAGCCGGGAGGCGGGAAGGTGCAGATAATTAAT
AAGAAGCTGGATCTTAGCAACGTCCAGTCCAAGTGTGGCTCAAAGGATAATATCAA
ACACGTCCCGGGAGGCGGCAGTGTGCAAATAGTCTACAAACCAGTTGACCTGAGCA
AGGTGACCTCCAAGTGTGGCTCATTAGGCAACATCCATCATAAACCAGGAGGTGGC
CAGGTGGAAGTAAAATCTGAGAAGCTTGACTTCAAGGACAGAGTCCAGTCGAAGAT
TGGGTCCCTGGACAATATCACCCACGTCCCTGGCGGAGGAAATAAAAAGATTGAAA
CCCACAAGCTGACCTTCCGCGAGAACGCCAAAGCCAAGACAGACCACGGGGCGGA
GATCGTGTACAAGTCGCCAGTGGTGTCTGGGGACACGTCTCCACGGCATCTCAGCAA
TGTCTCCTCCACCGGCAGCATCGACATGGTAGACTCGCCCCAGCTCGCCACGCTAGC
TGACGAGGTGTCTGCCTCCCTGGCCAAGCAGGGTTTGTAG

Mutant Tau P301L

ATGGCTGAGCCCCGCCAGGAGTTCGAAGTGATGGAAGATCACGCTGGGACGTACGG
GTTGGGGGACAGGAAAGATCAGGGGGGCTACACCATGCACCAAGACCAAGAGGGT
GACACGGACGCTGGCCTGAAAGAATCTCCCCTGCAGACCCCCACTGAGGACGGATC
TGAGGAACCGGGCTCTGAAACCTCTGATGCTAAGAGCACTCCAACAGCGGAAGATG
TGACAGCACCCTTAGTGGATGAGGGAGCTCCCGGCAAGCAGGCTGCCGCGCAGCCC
CACACGGAGATCCCAGAAGGAACCACAGCTGAAGAAGCAGGCATTGGAGACACCC
CCAGCCTGGAAGACGAAGCTGCTGGTCACGTGACCCAAGCTCGCATGGTCAGTAAA
AGCAAAGACGGGACTGGAAGCGATGACAAAAAAGCCAAGGGGGCTGATGGTAAAA
CGAAGATCGCCACACCGCGGGGAGCAGCCCCTCCAGGCCAGAAGGGCCAGGCCAA
CGCCACCAGGATTCCAGCAAAAACCCCGCCCGCTCCAAAGACACCACCCAGCTCTG
GTGAACCTCCAAAATCAGGGGATCGCAGCGGCTACAGCAGCCCCGGCTCCCCAGGC
ACTCCCGGCAGCCGCTCCCGCACCCCGTCCCTTCCAACCCCACCCACCCGGGAGCCC
AAGAAGGTGGCAGTGGTCCGTACTCCACCCAAGTCGCCGTCTTCCGCCAAGAGCCG
CCTGCAGACAGCCCCCGTGCCCATGCCAGACCTGAAGAATGTCAAGTCCAAGATCG
GCTCCACTGAGAACCTGAAGCACCAGCCGGGAGGCGGGAAGGTGCAGATAATTAAT
AAGAAGCTGGATCTTAGCAACGTCCAGTCCAAGTGTGGCTCAAAGGATAATATCAA
ACACGTCCTGGGAGGCGGCAGTGTGCAAATAGTCTACAAACCAGTTGACCTGAGCA
AGGTGACCTCCAAGTGTGGCTCATTAGGCAACATCCATCATAAACCAGGAGGTGGC
CAGGTGGAAGTAAAATCTGAGAAGCTTGACTTCAAGGACAGAGTCCAGTCGAAGAT
TGGGTCCCTGGACAATATCACCCACGTCCCTGGCGGAGGAAATAAAAAGATTGAAA
CCCACAAGCTGACCTTCCGCGAGAACGCCAAAGCCAAGACAGACCACGGGGCGGA
GATCGTGTACAAGTCGCCAGTGGTGTCTGGGGACACGTCTCCACGGCATCTCAGCAA
TGTCTCCTCCACCGGCAGCATCGACATGGTAGACTCGCCCCAGCTCGCCACGCTAGC
TGACGAGGTGTCTGCCTCCCTGGCCAAGCAGGGTTTGTAG

Control

ATGGCTGAGCCCCGCCAGGAGTTCGAAGTGATGGAAGATCACGCTGGGACGTACGG
GTTGGGGGACAGGAAAGATCAGGGGGGCTACACCATGCACTAAGACCAAGAGGGT
GACACGGACGCTGGCCTGAAAGAATCTCCCCTGCAGACCCCCACTGAGGACGGATC
TGAGGAACCGGGCTCTGAAACCTCTGATGCTAAGAGCACTCCAACAGCGGAAGATG
TGACAGCACCCTTAGTGGATGAGGGAGCTCCCGGCAAGCAGGCTGCCGCGCAGCCC
CACACGGAGATCCCAGAAGGAACCACAGCTGAAGAAGCAGGCATTGGAGACACCC
CCAGCCTGGAAGACGAAGCTGCTGGTCACGTGACCCAAGCTCGCATGGTCAGTAAA
AGCAAAGACGGGACTGGAAGCGATGACAAAAAAGCCAAGGGGGCTGATGGTAAAA
CGAAGATCGCCACACCGCGGGGAGCAGCCCCTCCAGGCCAGAAGGGCCAGGCCAA
CGCCACCAGGATTCCAGCAAAAACCCCGCCCGCTCCAAAGACACCACCCAGCTCTG
GTGAACCTCCAAAATCAGGGGATCGCAGCGGCTACAGCAGCCCCGGCTCCCCAGGC
ACTCCCGGCAGCCGCTCCCGCACCCCGTCCCTTCCAACCCCACCCACCCGGGAGCCC
AAGAAGGTGGCAGTGGTCCGTACTCCACCCAAGTCGCCGTCTTCCGCCAAGAGCCG
CCTGCAGACAGCCCCCGTGCCCATGCCAGACCTGAAGAATGTCAAGTCCAAGATCG
GCTCCACTGAGAACCTGAAGCACCAGCCGGGAGGCGGGAAGGTGCAGATAATTAAT
AAGAAGCTGGATCTTAGCAACGTCCAGTCCAAGTGTGGCTCAAAGGATAATATCAA
ACACGTCCCGGGAGGCGGCAGTGTGCAAATAGTCTACAAACCAGTTGACCTGAGCA
AGGTGACCTCCAAGTGTGGCTCATTAGGCAACATCCATCATAAACCAGGAGGTGGC
CAGGTGGAAGTAAAATCTGAGAAGCTTGACTTCAAGGACAGAGTCCAGTCGAAGAT
TGGGTCCCTGGACAATATCACCCACGTCCCTGGCGGAGGAAATAAAAAGATTGAAA
CCCACAAGCTGACCTTCCGCGAGAACGCCAAAGCCAAGACAGACCACGGGGCGGA
GATCGTGTACAAGTCGCCAGTGGTGTCTGGGGACACGTCTCCACGGCATCTCAGCAA
TGTCTCCTCCACCGGCAGCATCGACATGGTAGACTCGCCCCAGCTCGCCACGCTAGC
TGACGAGGTGTCTGCCTCCCTGGCCAAGCAGGGTTTGTAG
